# The pain funding gap: A database analysis of pain research funding in Canada from 2008–2023

**DOI:** 10.1080/24740527.2025.2486835

**Published:** 2025-05-02

**Authors:** S. S. Abssy, R. Bosma, S. Miles, H. Clarke, M. Moayedi

**Affiliations:** a Centre for Multimodal Sensorimotor and Pain Research, Faculty of Dentistry, University of Toronto, Toronto, Ontario, Canada; b University of Toronto Centre for the Study of Pain, University of Toronto, Toronto, Ontario, Canada; c Toronto Academic Pain Medicine Institute, Women’s College Hospital, Toronto, Ontario, Canada; d Department of Anesthesia and Pain Management, Toronto General Hospital, University Health Network, Toronto, Ontario, Canada; e Department of Anesthesiology and Pain Medicine, Temerty Faculty of Medicine, University of Toronto, Toronto, Ontario, Canada

**Keywords:** Pain, research, funding, operational funds, acute pain, chronic pain, Canadian Institutes of Health Research

## Abstract

**Background:**

One in five Canadians experiences chronic pain, at a cost of $40.3 billion in 2019. Despite this significant burden, there are few effective treatments for pain. This gap has been recognized by Health Canada, which has put forth the *Action Plan for Pain in Canada*. Advancing our understanding of pain mechanisms and clinical trials to identify novel therapeutics are essential to address this treatment gap. However, it remains unknown whether the recommendations of the *Action Plan* have increased research investments.

**Methods:**

We investigate research investments in pain by the Canadian Institutes of Health Research (CIHR) based on publicly available data. We performed a systematic database search focused on operating funds from competitions between 2008 and 2023 and tabulated pain funding as a proportion of total CIHR operational funds granted each year. Next, we examined the proportion of pain funding across CIHR institutes aggregated across funding years.

**Results:**

We identified 20,126 operational grants, of which 459 were pain focused. The highest level of pain funding was 3.32% in 2019, and the average (SD) was 2.13% (0.70%). Funding was stagnant from 2008 to 2023 (*R*^2^ = 0.10, *P* = 0.23). The Institute of Musculoskeletal Health and Arthritis allocated the largest proportion of funding to pain research (11.40%). Eight of the 13 institutes allocated less than 1% of their operating funds to pain research.

**Interpretation:**

In sum, CIHR pain research funding does not match the socioeconomic burden posed by pain. We propose three action items to improve pain research funding and to ultimately relieve the burden of pain in Canada.

## RÉSUMÉ

**Contexte :** Un Canadien sur cinq souffre de douleur chronique, ce qui représentait un coût de 40,3 milliards de dollars en 2019. Malgré ce fardeau important, ls traitements efficaces contre la douleur demeurent peu nombreux. Cette lacune a été reconnue par Santé Canada, qui a publié le Plan d’action contre la douleur au Canada. Pour combler cette lacune, il est essentiel de mieux comprendre les mécanismes de la douleur et de mener des essais cliniques pour mettre au point de nouveaux traitements. Cependant, on ne sait pas si les recommandations du plan d’action ont conduit à une augmentation des investissements dans la recherche.

**Méthodes :** Nous avons examiné les investissements en recherche sur la douleur par les Instituts de recherche en santé du Canada (IRSC), à partir de données accessibles au public. Une recherche systématique a été effectuée dans leur base de données, axée sur les fonds de fonctionnement des compétitions entre 2008 et 2023, et nous avons tabulé le fnancement de la douleur en proportion du total des fonds de fonctionnement des IRSC alloués chaque année. Nous avons ensuite analysé la proportion du financement destiné à la douleur au sein des instituts des IRSC, regroupés par année de financement.

**Résultats :** Nous avons recensé 20 126 subventions opérationnelles, dont 459 étaient consacrées à la douleur. Le pourcentage le plus élevé de financement de la douleur était de 3,32 % en 2019, et la moyenne (écart-type) était de 2,13 % (0,70 %). Le financement est resté stable de 2008 à 2023 (R2 = 0,10, P = 0,23). L’Institut de la santé musculosquelettique et de l’arthrite a alloué la plus grande partie de son financement à la recherche sur la douleur (11,40 %). Huit des 13 instituts ont alloué moins de 1 % de leurs fonds de fonctionnement à la recherche sur la douleur.

**Interprétation :** En somme, le financement de la recherche sur la douleur des IRSC ne correspond pas au fardeau socio-économique engendré par la douleur. Nous proposons trois actions pour améliorer le financement de la recherche sur la douleur et, en définitive, alléger le fardeau de la douleur au Canada.

## Introduction

Chronic pain impacts one in five Canadians,^1–3^ costs $40.4 billion per year in Canada,^1^ and is a top contributor to the global burden of disease.^4,5^ Pain is difficult to treat because it results from a complex interplay of biological, psychological, and social factors^6^; is caused by multiple pathophysiological mechanisms^7–9^; and can be a symptom of many disparate diseases or a disease in its own right.^10^ Despite its significant burden, our understanding of pain is rudimentary, and there are few efficacious treatments for many pain conditions.^11^ Given the prevalence, cost, and a lack of effective treatments, a focus on pain research is urgently needed.

Canada is a global leader in pain research, producing paradigm-shifting advances, despite limited funding. Recent actions from national and international organizations have made pain a policy priority. The World Health Organization has recognized some chronic pain as primary diseases,^10,12,13^ improving the visibility of chronic pain and providing a common nomenclature for pain research.^14^ Health Canada set a goal to “support pain research and strengthen related infrastructure” in its 2021 *Action Plan for Pain in Canada*.^1^ However, whether the recommendations from the *Action Plan* have resulted in greater pain research funding in Canada remains unclear.

The largest funding source for health research in Canada is the Canadian Institutes of Health Research (CIHR), which invests ~$1.2 billion in health research annually.^15^ CIHR comprises 13 research institutes, none of which has a mandate to study pain, but pain is relevant to several of the institutes (e.g., the Institute of Musculoskeletal Health and Arthritis, the Institutes of Neurosciences, Mental Health and Addiction).^15^ The goal of this study was to explore the level of pain research funding in Canada by describing trends in CIHR funding over the past 15 years and by delineating which CIHR institutes spend the highest percentage of their research funding on pain research. Outcomes from this study will identify gaps in funding policy and promote targeted pain funding in Canada.

## Methods

Grant funding data were pulled from the CIHR Funding Decisions Database^16^ (CFDD). We limited our search to grants that funded operations (i.e., excluding salary awards, studentships, etc.). We sought to determine the proportion of funding for pain research compared to the overall CIHR funding envelope for each year between 2008 and 2023. In a second analysis, we compared operational pain funding to overall operational funding aggregated from 2008 to 2023 for each of the 13 CIHR institutes, as well as grants that were not assigned to an institute. The date range used was based on available data that is fully captured on the CIHR Funding Decisions Database’s graphical user interface (GUI).

### Screening for Operating Grants

#### Preparing the Full Operating Grant Dataset

All grant data were sourced from the CFDD. Note that the database can be accessed through a GUI that allows for keyword selection and institute(s) that funded grants; the CFDD downloadable document does not include these data. Therefore, any keyword searches and institute-specific searches were performed in the GUI.

To screen for operating grants and grants that support research operating activities, we filtered grants by country/region to limit to Canadian grants and by program family to include operating grants, undefined grants, directed grants, randomized controlled trials, and miscellaneous programs; we excluded all other types of funding including studentships, salary awards, summer student research funding, and exchange programs.

#### Institute-Specific Filtering

To identify CIHR institute-specific funding data, all operational funding grants were labeled and extracted based on the reported CIHR institute administering the funds. This allowed us to determine the overall funding for each institute, which was later used as a reference to compare funding allocations to pain research.

#### Secondary Screening

Because we were only interested in CIHR grants that provided research operating funds, we performed a secondary screening using exclusion terms applied to the (1) program name and (2) peer review committee of each grant. Program name refers to the specific funding initiative or grant program under which a project is funded, and the peer review committee refers to a group of experts responsible for evaluating and scoring grant applications within a specific research area or field. The exclusion terms used were “animal care” (which captures grants related to animal welfare, rather than human health–related research), “award,” “Café Scientifique,” “dissemination,” “fellow,” “LOI,” “meeting,” “mentor,” “planning,” “prize,” “special case,” “summer,” “synapse,” “training,” “Tr. Gr.” (indicating training grant), and “travel.” We visually inspected the remaining grants to ensure that they met inclusion criteria. Lastly, grants were limited to the years 2008 to 2023, because 2007 and 2024 were not fully captured by the CFDD. All excluded grants are listed in Table S1.

### Identifying Pain-Specific Operating Grants

#### Identifying Pain Grants

To identify pain-specific grants, we first utilized keywords. In the CFDD GUI, CIHR defines keywords as “descriptors that provide the necessary information for assigning reviewers with the appropriate expertise to an application.” We used the keywords “pain” and “*douleur*” to identify pain grants. Next, to ensure that we captured pain grants that were not denoted with based on our keyword criteria, we performed a title search in the GUI. After deduplication, all grants that contained the words “pain” and “*douleur*” in the title were identified and manually screened for relevance. Lastly, we created a pain funding–specific database using the same approach described above, using keywords to identify pain-related grants.

### Outcome Measures

Once our complete operating activities grant data set was established, we conducted a series of analyses comparing pain funding to total funding. We pooled funding amounts by year. We also pooled funding amounts across all years by institute. In all instances, we utilized the “CIHR contribution” amount associated to each grant to define how much operating funding that grant received because CIHR-funded “equipment contributions” fall outside of the scope of operating activities and were not included in our analyses.

### Statistical Analysis

To determine whether there were changes to CIHR operational pain funding from 2008 to 2023, we performed a simple linear regression with proportion of funding as the dependent variable, and year of funding as the independent variable in Prism. Significance was set at *P* < 0.05.

## Results

**Figure 1.**
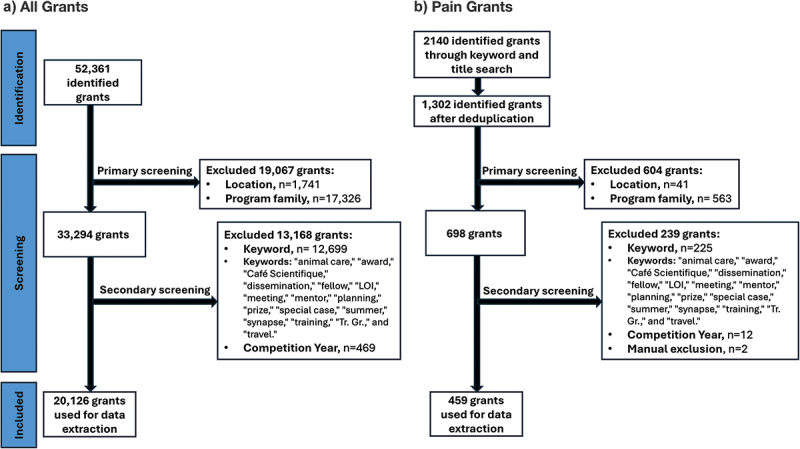
Preferred Reporting Items for Systematic reviews and Meta-Analyses flow diagrams of CIHR-funded operating grants from 2008 to 2023. (a) Grants from all areas of health research and (b) pain-focused research based on the CIHR Funding Decisions Database. All grants underwent primary screening for location of funding (grants held in Canada), program family (operational grant funding), and secondary screening (based on keywords). Pain grants also underwent manual screening in the secondary screening stage.

We identified 52,361 grants in the CFDD. We excluded 19,067 grants in the first screening phase based on location (*n* = 1,741) and program family (*n* = 17,326). We excluded 13,168 grants in the second screening phase through keyword exclusion (*n* = 12,699) and competition year (*n* = 469), leaving 20,126 operational grants in our analysis (see Figure 1a, Table S2).

To identify pain-specific operational grants, 2140 pain grants were first identified using the keyword search and title search using “pain” and “*douleur*.” After deduplication, 1,302 pain grants remained (see Figure 1b). We then excluded 604 grants based on location (*n* = 41) and program family (*n* = 563). We excluded an additional 239 grants in the second screening phase through keyword exclusion (*n* = 225), competition year (*n* = 12), and manual exclusion (*n* = 2), leaving 459 pain grants and 19,667 non-pain grants in our analysis.

**Figure 2.**
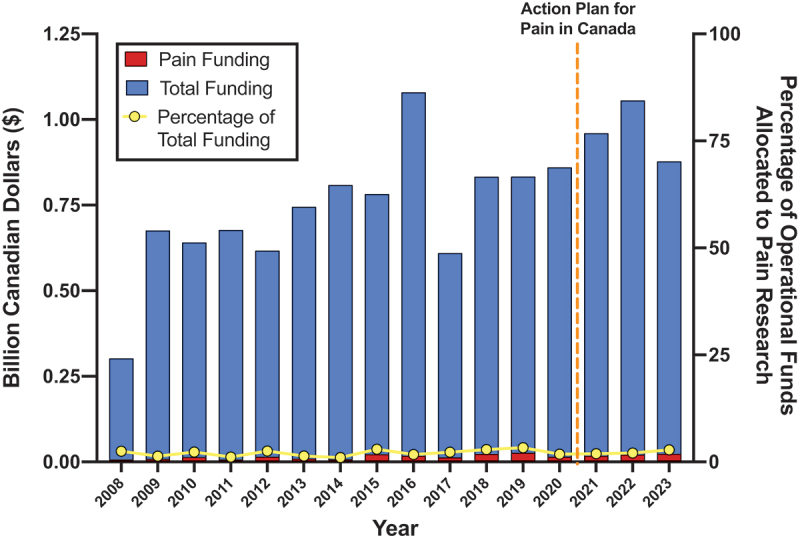
Percentage of CIHR operating grants allocated to pain research for 2008 to 2023. Pain funding is shown in red, and total funding is shown in blue. Both pain funding and total funding are measured on the left *y* axis. Percentage of total funding that represents pain funding is shown in yellow and is measured on the right *y* axis. The dashed orange line represents the date of the *Action Plan for Pain in Canada.*

Based on our taxonomic approach, we calculated CIHR funding for operating grants for each year between 2008 and 2023 (see Figure 2, Table S3). The percentage of total CIHR contributions showed substantial year-to-year variability with an average of 2.13% and standard deviation of 0.70%. The lowest funding percentage was observed in 2014 at 1.02%, and the highest occurred in 2019 at 3.32%. Fluctuations in funding included a notable increase from $8.1 million in 2014 (1.02% of total funding) to $22.7 million in 2015 (2.99% of total funding), followed by a decrease to $15.4 million in 2020 (1.82% of total funding). Consistent upward trends were noted from 2016 to 2019 and 2020 to 2023 (1.73% to 3.32% and 1.82% to 2.81%, respectively).

**Figure 3.**
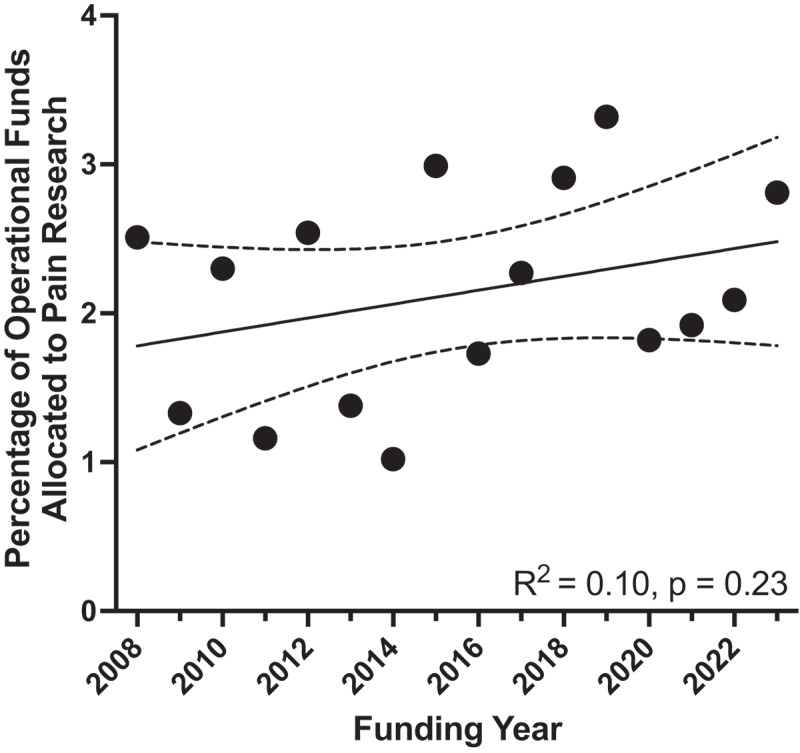
Percentage of annual CIHR operational funding for pain research. Pain funding (as a percentage of total operational funds) increased at a slope of 0.047%/year, with an *R*^2 ^= 0.10, *P* = 0.23. The line of best fit (solid line) and 95% confidence intervals (dashed lines) are plotted.

We performed a simple linear regression to determine whether there were statistically significant increases in the proportion of pain funding to total CIHR operational funding from 2008 to 2023. We found that funding did not increase significantly, with an increase of 0.047% per year (*R*^2^ = 0.102, F_1,14_ = 1.590, *P* = 0.23; Figure 3).

### Institute-Specific Pain Analysis

**Figure 4.**
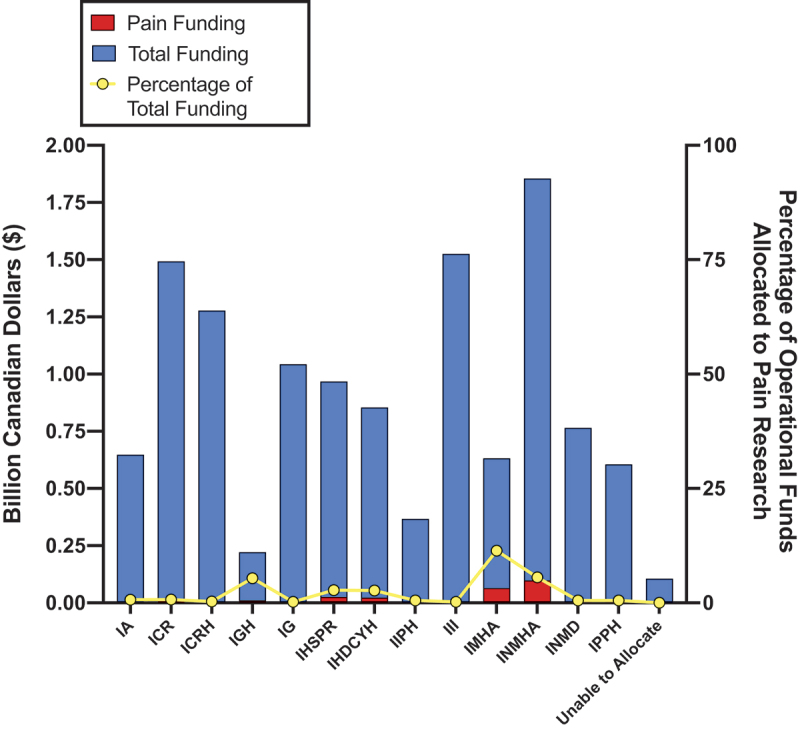
Operating activities funded through each CIHR institute aggregated from 2008 to 2023. Pain funding is shown in red, and total funding is shown in blue. Both pain funding and total funding (in billions of Canadian dollars) are measured on the left *y* axis. The proportion of pain funding to total operational funding (in percentages) is shown in yellow and is measured on the right *y* axis. Unable to Allocate indicates all grants that lack institute specific funding. IA = Institute of Aging; ICR = Institute of Cancer Research; ICRH = Institute of Circulatory and Respiratory Health; IG = Institute of Genetics; IGH = Institute of Gender and Health; IHDCYH = Institute of Human Development, Child and Youth Health; IHSPR = Institute of Health Services and Policy Research; III = Institute of Infection and Immunity; IIPH = Institute of Indigenous People’s Health; IMHA = Institute of Musculoskeletal Health and Arthritis; INMD = Institute of Nutrition, Metabolism and Diabetes; INMHA = Institute of Neurosciences, Mental Health and Addiction; IPPH = Institute of Population and Public Health.

Utilizing our taxonomic approach, we calculated CIHR institute-specific funding for operational grants (see Figure 4, Table S4). From 2008 to 2023 the Institute of Musculoskeletal Health and Arthritis (IMHA) allocated the highest percentage of its operating funding for pain research at 11.40%, amounting to $567,137,897. The Institutes of Neurosciences, Mental Health and Addiction (INMHA) contributed 5.58% of their funding for pain research, which represents the largest absolute amount of institute-specific pain funding at $98,118,070. The Institute of Gender and Health (IGH) followed INMHA and IMHA closely with 5.39% of its budget directed toward pain research, which amounted to $11,326,508.

In terms of absolute amount of pain-specific funding, INMHA and IMHA were the top two funders. The next top funders of pain research were the Institutes of Health Services and Policy Research ($26,072,404; 2.77%); Human Development, Child and Youth Health ($22,611,476; 2.72%); the Institute of Gender and Health ($11,326,508, 5.39%); and Cancer Research ($10,468,753; 0.71%).

In contrast, institutes such as Aging (0.68%, $4,340,521); Circulatory and Respiratory Health (0.30%, $3,884,508); Genetics (0.23%, $2,419,506); Indigenous Peoples’ Health (0.53%, $1,939,164); Infection and Immunity (0.21%, $3,162,725); Nutrition, Metabolism, and Diabetes (0.51%, $3,912,649); and Population and Public Health (0.54%, $3,258,166) each contributed less than 1% in their total operating funds, with absolute funding amounts below $5 million. No grants under the “unable to allocate” category supported pain funding. Full institute-specific funding distributions are provided in Table S4.

### Interpretation

Using CIHR’s publicly available Funding Decisions Database, we identified that pain grants made up 459 of 20,126 operating grants funded by CIHR between 2008 and 2023 (see Table S2). As shown in Figure 2, pain grants received an average of 2.13% of operational funding each year, which did not change over the period (Figure 3), indicating that Health Canada’s goal to “support pain research and strengthen related infrastructure”^1^ has not translated to increased CIHR funding for pain-specific research over this period of time. Based on an analysis of funding based on CIHR Institutes between 2008 and 2023 (Figure 4, Table S4), the IMHA allocated the highest percent of its operating funding for pain research (11.40%), followed by the Institutes of Neurosciences, Mental Health and Addiction (5.58%) and Institute of Gender and Health (5.39%). The Institute of Neurosciences, Mental Health and Addiction contributed the highest absolute amount of pain-specific funding (roughly $98 million over 15 years or 30 grant review cycles, averaging $6.5 million per year).

Although pain poses the largest health-related burden in Canada, affecting one in five Canadians,^1–3^ we found that only 2% of federal health research funding is directed toward pain research. In 2023 CIHR funded just over $24 million in pain research, which amounts to roughly $3 for each of the 8 million Canadians experiencing chronic pain. Without changes to pain research funding in Canada, this mismatch between the societal burden of pain and its research funding level will only worsen as our aging population increases, as well as the number of Canadians impacted by pain. In many ways, we cannot afford this mismatch between the burden of pain and dedicated research funding. The economic burden of pain in Canada is $40.4 billion per year,^1^ pain is the leading cause of disability in the working-age population,^17^ and the few effective pain treatments that exist tend to be very burdensome to the already strained health care system.^18,19^ Yet even small improvements to chronic pain prevention and treatment have the potential to lead to substantial savings. Even a 1% decrease in the number of Canadians living with chronic pain could save $400 million (2020 CA$) per year.^1^

Our findings align with previous investigations of pain funding in Canada. For example, in 2009, Lynch and colleagues surveyed members of the Canadian Pain Society about their research funding. Based on the 79 responses, they found that the bulk of pain funding was from CIHR. Our study goes further and uses objective data from CIHR to determine what proportion of their funding is allocated to pain research between 2008 and 2023 and which institutes funded this research. Our findings highlight chronic underfunding of the pain.

Clearly, research and innovation are needed to drive new therapeutic advances and treatment paradigms to address this societal burden. Such innovation can only be achieved through targeted funding, which has been successful in several other fields. In the 1980s, governments and organizations like the National Institutes of Health and the Bill & Melinda Gates Foundation committed significant funding to HIV/AIDS research, leading to the development of antiretroviral therapy, which transformed HIV/AIDS from a fatal disease into a manageable chronic condition.^20^ In 2016 the Cancer Moonshot Initiative allocated $1.8 billion to accelerate cancer research, leading to breakthroughs in immunotherapy, personalized medicine, early detection technologies, and CAR-T therapy, a revolutionary cancer treatment using a patient’s own immune cells to fight the disease.^21,22^ In 2020, billions of dollars in targeted funding accelerated the development, production, and distribution of COVID-19 vaccines in an unprecedented manner and had widespread impact as discoveries continue to be transferred to other domains.^23,24^

Like HIV/AIDS, cancer, and COVID-19, pain is a complex phenomenon that needs complex solutions across the spectrum of basic science to clinical research. Despite this need to bring together researchers across disciplines, there is no “home” for pain research in Canada. Most CIHR funding for pain research comes from the INMHA and the IMHA, neither of which includes pain in their institutional mandate (although alleviating pain is a priority area for IMHA). In the United States, the need for a multidisciplinary research initiative to improve pain prevention and treatment was recognized by the National Institutes of Health’s Heal Initiative.^25^ This research effort funded 1800 research projects in 2024 to improve “the understanding, management, and treatment of pain” and “the prevention and treatment of opioid misuse, addiction, and overdose.”^25^ In contrast, the Canadian funding ecosystem for pain research remains fragmented, with no targeted or sustained funding for pain research and no dedicated institute to support pain research (e.g., to identify research priorities, fund pain research, support knowledge translation), highlighting the need for a coordinated Canadian pain research agenda.^1,26^

Despite this lack of a pain research “home” and relatively low funding for pain research, Canada is a world leader in pain research.^27,28^ Work is already being done to integrate many of the recommendations in the 2021 *Action Plan for Pain in Canada* into science funding policy. Continued meaningful investment in targeted research funding is required to strengthen the Canadian pain research ecosystem and enact change. This could be catalyzed by prioritizing pain research across all institutes and establishing an expert peer review panel for pain-related grants. According to the CIHR website, “Priority-driven research refers to initiatives created by the Government of Canada to investigate pressing health issues that are of strategic importance to our country.”^29^ Therefore, despite the *Action Plan*’s recommendations (in particular, Goal 4), and seven CIHR cycles since its publication, the federal government has not set pain as a research priority. In fact, the CIHR action plan (published in 2021) does not include pain research as a priority.

Institutes can also set their own priorities, however; as shown in our study, pain research spans multiple institutes. Each institute has published their 5-year strategic plans (e.g., 2021–2026, 2022–2027, 2023–2028, or 2024–2029), but only one institute, the IMHA, includes “relieving the burden of chronic pain.” However, in terms of operational funds, these are limited to bridging grants funded through the Priority Announcements for Project Grant Competitions ($100,000/cycle). We are pleased to see this commitment and commend the institute for directly addressing the impact of pain. They have also announced team grants (~$5.6 million over 5 years) to focus on research evaluating models of care relating to painful conditions. However, given the limited nature of the investment, and the limited scope of funding, we are concerned about how these priorities can be scaled up to meet the population need posed by pain in Canada.

No other institute discusses pain. For example, the Institute of Aging discusses chronic diseases and specifically mentions dementia and Alzheimer’s but not pain. This is surprising because the aging population was noted as a key factor in the increasing burden of pain in Canada. The Institute of Gender and Health cite recent discoveries of sex differences in pain mechanisms but do not discuss any focus on pain or further investment in this research domain. The Institute of Health Services and Policy Research discuss how delays in care can lead to unnecessary and burdensome pain but does not discuss how to alleviate this pain. The INMHA led an initiative where CIHR funded 15 operating grants as part of a “rapid response” initiative on the “evaluation of interventions to address the opioid crisis,” each capped at $100,000.^30,31^ Of these, 4 were directly related to pain, whereas 11 were focused on opioid-use disorder. No other institutes mention pain, discuss the burden of chronic pain, or set pain research as a priority or as part of their strategic plans.

Another way of improving the funding landscape for pain research in Canada (and to follow the proposed goals of the *Action Plan for Pain* in Canada) would be to establish a pain-focused peer review committee. CIHR periodically evaluates peer review committees. Priority announcements also have specialized, expert peer review committees. A pain-focused peer review committee would mitigate the current requirement for pain researchers to find the best possible fit for their research proposals and often have non-experts review their grants. Currently, five different panels have pain outlined in their mandates: behavioral sciences–A, behavioral sciences–C, biological and clinical aspects of aging, systems and circuit neurosciences, and social dimensions of aging. In contrast, there are multiple panels focused on particular systems (e.g., cardiovascular system–A, B, and C; cancer biology, respiratory system). At face value, it may appear that there are several panels for neurosciences/neurological sciences, but these represent multiple sensory, cognitive, limbic, and motor systems and the broad array of diseases that can affect each of these systems. Therefore, in an effort to prioritize pain, a pain-focused peer review panel would greatly improve the caliber of reviews and the number of high-quality pain-focused grants funded per cycle.

Furthermore, establishing a set of common data elements to be collected in all human pain research paired with open science initiatives will create the infrastructure to expand the scope and breadth of pain research and mobilize collaborative efforts of pain research across Canada.

One noted limitation of this study is that we have only described research funding from CIHR. Although CIHR is Canada’s primary source of funding for health research, provincial funding bodies and nongovernmental organizations (e.g., Arthritis Society Canada, Canadian Cancer Society) also modestly contribute to the pain research funding ecosystem. It was not possible to quantify trends in pain research funding from these sources because they are not readily available. A second limitation is that our strategy for identifying pain research depends on the use of the terms “pain” or “*douleur*” in the title or keywords by grant applicants. We recommend that all future grants related to pain (whether fundamental biology, mechanisms, care or health systems) use the keyword “pain/*douleur*” to increase visibility and for systematic categorization.

Overall, we found that 2.13% of CIHR’s operational research funding was directed toward pain research, a percentage which did not increase substantially over the study period, despite an increased prioritization of chronic pain research at the federal level. Pain research funding was awarded by several CIHR institutes, with the largest relative and absolute amounts coming from the IMHA and the INMHA, even though neither of these institutes are mandated to fund pain research. Because chronic pain is now recognized as a disease in its own right, a plan for targeted and sustained research funding is instrumental to drive innovation that will transform pain management and reduce the number one driver of disability in Canada.

## Supplementary Material

Table_S2.xlsx

SupplementalMaterial_R1_Final.docx

Table_S1.xlsx

## References

[1] Health Canada. An Action Plan for Pain in Canada. Online: Government of Canada; Updated 2021 May [accessed 2025 Jan 16]. https://www.canada.ca/content/dam/hc-sc/documents/corporate/about-health-canada/public-engagement/external-advisory-bodies/canadian-pain-task-force/report-2021-rapport/report-rapport-2021-eng.pdf.

[2] Schopflocher D, Taenzer P, Jovey R. The prevalence of chronic pain in Canada. Pain Res Manag. 2011 Nov-Dec;16(6):445–9. doi:10.1155/2011/876306. 22184555 PMC3298051

[3] Shupler MS, Kramer JK, Cragg JJ, Jutzeler CR, Whitehurst DGT. Pan-Canadian estimates of chronic pain prevalence from 2000 to 2014: a repeated cross-sectional survey analysis. J Pain. 2019 May;20(5):557–65. doi:10.1016/j.jpain.2018.10.010. 30503860

[4] Briggs AM, Woolf AD, Dreinhöfer K, Homb N, Hoy DG, Kopansky-Giles D, Åkesson K, March L. Reducing the global burden of musculoskeletal conditions. Bull World Health Organ. 2018 May 01;96(5):366–68. doi:10.2471/BLT.17.204891. 29875522 PMC5985424

[5] Rice ASC, Smith BH, Blyth FM. Pain and the global burden of disease. Pain. 2016 Apr;157(4):791–96. doi:10.1097/j.pain.0000000000000454. 26670465

[6] Gatchel RJ, Peng YB, Peters ML, Fuchs PN, Turk DC. The biopsychosocial approach to chronic pain: scientific advances and future directions. Psychol Bull. 2007 Jul;133(4):581–624. doi:10.1037/0033-2909.133.4.581. 17592957

[7] Nijs J, George SZ, Clauw DJ, Fernández-de-las-Peñas C, Kosek E, Ickmans K, Fernández-Carnero J, Polli A, Kapreli E, Huysmans E, et al. Central sensitisation in chronic pain conditions: latest discoveries and their potential for precision medicine. Lancet Rheumatol. 2021 May;3(5):e383–e392. doi:10.1016/S2665-9913(21)00032-1. 38279393

[8] Finnerup NB, Kuner R, Jensen TS. Neuropathic pain: from mechanisms to treatment. Physiol Rev. 2021 Jan 01;101(1):259–301. doi:10.1152/physrev.00045.2019. 32584191

[9] Puntillo F, Giglio M, Paladini A, Perchiazzi G, Viswanath O, Urits I, Sabbà C, Varrassi G, Brienza N. Pathophysiology of musculoskeletal pain: a narrative review. Ther Adv Musculoskelet Dis. 2021;13:1759720X21995067. doi:10.1177/1759720X21995067. PMC7934019 33737965

[10] Treede RD, Rief W, Barke A, Aziz Q, Bennett MI, Benoliel R, Cohen M, Evers S, Finnerup NB, First MB, et al. A classification of chronic pain for ICD-11. Pain. 2015 Jun;156(6):1003–07. doi:10.1097/j.pain.0000000000000160. 25844555 PMC4450869

[11] Skelly AC, Chou R, Dettori JR, Turner JA. Noninvasive nonpharmacological treatment for chronic pain: a systematic review update. Rockville (MD). Agency for Healthcare Research and Quality (US); 2020 Apr [accessed 2024 Oct 31]. https://www.ncbi.nlm.nih.gov/books/NBK556228/. 32338846

[12] World Health Organization. ICD-11 Implementation. [accessed 2024 Oct 31]. https://www.who.int/standards/classifications/frequently-asked-questions/icd-11-implementation.

[13] World Health Organization. Frozen version of ICD11 for implementation. http://www.who.int/classifications/icd/en/.

[14] Barke A, Korwisi B, Jakob R, Konstanjsek N, Rief W, Treede RD. Classification of chronic pain for the International Classification of Diseases (ICD-11): results of the 2017 international World Health Organization field testing. Pain. 2022 Feb 01;163(2):e310–e318. doi:10.1097/j.pain.0000000000002287. 33863861 PMC8756346

[15] Canadian Institutes of Health Research. Canadian Institutes of Health Research. [accessed 2024 Oct 31]. https://cihr-irsc.gc.ca/e/193.html.

[16] Canadian Institutes of Health Research. Funding Decisions Database. Updated 2023 Dec 15. [accessed 2025 January 21]. https://webapps.cihr-irsc.gc.ca/decisions/p/main.html

[17] Statistics Canada. Canadian survey on disability, 2017 to 2022. The Daily; 2023.

[18] Park PW, Dryer RD, Hegeman-Dingle R, Mardekian J, Zlateva G, Wolff GG, Lamerato LE. Cost burden of chronic pain patients in a large integrated delivery system in the United States. Pain Pract. 2016 Nov;16(8):1001–11. doi:10.1111/papr.12357. 26443292

[19] Collard VEJ, Moore C, Nichols V, Ellard DR, Patel S, Sandhu H, Parsons H, Sharma U, Underwood M, Madan J, et al. Challenges and visions for managing pain-related insomnia in primary care using the hybrid CBT approach: a small-scale qualitative interview study with GPs, nurses, and practice managers. BMC Fam Pract. 2021 Oct 20;22(1):210. doi:10.1186/s12875-021-01552-3. 34666682 PMC8527665

[20] National Institutes of Health. NIH office of AIDS research: 35 years of advancing HIV research. [accessed 2024 Nov 5]. https://oar.nih.gov/about/history/oar-35-years.

[21] Annapragada A, Sikora AG, Marathe H, Liu S, Demetriou M, Fong L, Gao J, Kufe D, Morris ZS, Vilar E, et al. The cancer moonshot immuno-oncology translational network at 5: accelerating cancer immunotherapies. J Natl Cancer Inst. 2023 Nov 08;115(11):1262–70. doi:10.1093/jnci/djad151. 37572314 PMC10637038

[22] National Institutes of Health. Cancer Moonshot- Recent Fiscal Year Funding. [accessed 2024 Nov 5]. https://www.cancer.gov/about-nci/budget/fact-book/cancer-moonshot.

[23] Slaoui M, Hepburn M. Developing safe and effective Covid vaccines - Operation warp speed’s strategy and approach. N Engl J Med. 2020 Oct 29;383(18):1701–03. doi:10.1056/NEJMp2027405. 32846056

[24] Meslé MMI, Brown J, Mook P, Katz MA, Hagan J, Pastore R, Benka B, Redlberger-Fritz M, Bossuyt N, Stouten V, et al. Estimated number of lives directly saved by COVID-19 vaccination programmes in the WHO European Region from December, 2020, to March, 2023: a retrospective surveillance study. Lancet Respir Med. 2024 Sep;12(9):714–27. doi:10.1016/S2213-2600(24)00179-6. 39127051

[25] National Institutes of Health. About the NIH HEAL Initiative. [accessed 2024 Oct 30]. https://heal.nih.gov/about.

[26] Campbell F, Hudspith M, Anderson M, Choinieère M, El-Gabalawy H, Laliberteé J, Swidrovich J, Wilhelm L. Chronic Pain in Canada: Laying a Foundation for Action. Ottawa: Her Majesty the Queen in Right of Canada, as represented by the Minister of Health, 2019; [accessed 2025 Jan 16]. https://www.canada.ca/content/dam/hc-sc/documents/corporate/about-health-canada/public-engagement/external-advisory-bodies/canadian-pain-task-force/report-2019/canadian-pain-task-force-June-2019-report-en.pdf

[27] Chen L, Li N, Zhang Y. High-impact papers in the field of anesthesiology: a 10-year cross-sectional study. *Can J Anaesth*. Feb 2023;70(2):183–90. doi:10.1007/s12630-022-02363-5. 36418743 PMC9684867

[28] Robert C, Wilson CS, Donnadieu S, Gaudy JF, Arreto CD. Evolution of the scientific literature on pain from 1976 to 2007. *Pain Med*. May 2010;11(5):670–84. doi:10.1111/j.1526-4637.2010.00816.x. 20202144

[29] Canadian Institutes of Health Research. Initiatives: Research In Priority Areas. Updated August 20, 2024 [Accessed January 11, 2025]. https://cihr-irsc.gc.ca/e/50077.html.

[30] Canadian Institutes of Health Research. Funded Projects: Evaluation of Interventions to Address the Opioid Crisis. Updated May 15, 2019 [Accessed January 16, 2025]. https://www.canada.ca/en/institutes-health-research/news/2019/05/funded-projects-evaluation-of-interventions-to-address-the-opioid-crisis.html.

[31] Canadian Institutes of Health Research. Neurosciences, Mental Health and Addiction Strategic Plan 2020-2022. Updated December 14, 2020 [Accessed January 9, 2025]. https://cihr-irsc.gc.ca/e/52253.html#4.1.1.1.

