# Correction Notice

**DOI:** 10.1080/24740527.2025.2600250

**Published:** 2026-01-14

**Article title**: The pain funding gap: A database analysis of pain research funding in Canada from 2008–2023

**Authors**: S. S. Abssy, R. Bosma, S. Miles, H. Clarke & M. Moayedi.

**Journal**: *Canadian Journal of Pain*

**Bibliometrics**: 9:1, 2486835

**DOI**: 10.1080/24740527.2025.2486835

After publication, the authors and Journal were alerted to concerns regarding the accuracy of the data shown in Figure 4, the interpretations being made based on publicly available information, and the conclusions reported in the article.

To ensure transparency with regards to what data and websites were accessed, and when the data reported in the article was collected, the authors are correcting their article by including the following:

- The incorrect citation for the source of the CIHR Funding Decisions Database has been corrected to: Canadian Institutes of Health Research. Funding Decisions Database. Updated December 15, 2023 [accessed 2025 Jan 21]. https://webapps.cihr-irsc.gc.ca/decisions/p/main.html
- The dates of access have been added to the relevant references
- The following references were added to the manuscript to support the statements made:
  - Canadian Institutes of Health Research. Funded Projects: Evaluation of Interventions to Address the Opioid Crisis. Updated May 15, 2019 [Accessed January 16, 2025]. https://www.canada.ca/en/institutes-health-research/news/2019/05/funded-projects-evaluation-of-interventions-to-address-the-opioid-crisis.html
  - Canadian Institutes of Health Research. Neurosciences, Mental Health and Addiction Strategic Plan 2020-2022. Updated December 14, 2020 [Accessed January 9, 2025]. https://cihr-irsc.gc.ca/e/52253.html

The Publisher in consultation with the Journal has confirmed that the data in Figure 4 and the article as a whole was deemed accurate at the time of writing and publication based on the then publicly available information.

*Footnote: all the authors approved the correction. The Editor-in-Chief recused himself from the investigation process and took on an advisory role.*

