# Supplementary material for: The pain funding gap: A database analysis of pain research funding in Canada from 2008–2023
Source: Can J Pain. 2025 May 2;9(1):2486835. doi: 10.1080/24740527.2025.2486835 (PMC12051529; doi:10.1080/24740527.2025.2486835)
Supplement: SupplementalMaterial_R1_Final.docx [file UCJP_A_2486835_SM2767.docx]

**Supplementary Materials for:**

**The Pain Funding Gap: A Database Analysis of Pain Research Funding in Canada from 2008-2023**

SS Abssy HBSc^1,2^, R Bosma PhD^2,3^, S Miles PhD^4^, H Clarke MD PhD*^2,5^, M Moayedi PhD*^1,2^

1. Centre for Multimodal Sensorimotor and Pain Research, Faculty of Dentistry, University of Toronto, Toronto, ON, Canada
2. University of Toronto Centre for the Study of Pain, University of Toronto, Toronto, ON, Canada
3. Toronto Academic Pain Medicine Institute, Women’s College Hospital, Toronto, ON, Canada
4. Department of Anesthesia and Pain Management, Toronto General Hospital, University Health Network, Toronto, ON, Canada
5. Department of Anesthesiology and Pain Medicine, Temerty Faculty of Medicine, University of Toronto, Toronto, ON, Canada

**Disclosures:** HC is President of the Canadian Pain Society (CPS), MM is Secretary of CPS and Councilor for the International Association for the Study of Pain. RB is the Chair of the Board of Directors for Pain Ontario.

**Funding Statement**: This work was funded by a Substance Use and Addictions Program Grant from Health Canada to HC.

***Corresponding Authors:**

Dr. Hance Clarke Dr. Massieh Moayedi

Department of Anesthesia Centre for Multimodal Sensorimotor

and Pain Management and Pain Research

Toronto General Hospital Faculty of Dentistry

University Health Network University of Toronto

200 Elizabeth Street 124 Edward Street
Toronto, ON Toronto, ON
Canada M5G 2C4 Canada M5G 1G6

Email: hance.clarke@uhn.ca Email: m.moayedi@utoronto.ca

**Table S1: List of excluded grants**

(See Appended)

**Table S2: List of included grants**

(See Appended)

**Table S3: Funding Summary from 2008-2023**

| **Competition**  **Year** | **Operational Grant Funding Envelope (CAD)** | **Total Operational Pain Funding (CAD)** | **Total Funding for All Other research Areas (CAD)** | **Percentage of Pain Funding (%)** |
| --- | --- | --- | --- | --- |
| 2008 | 295,337,218 | 7,405,902 | 287,931,316 | 2.51 |
| 2009 | 667,099,198 | 8,890,059 | 658,209,139 | 1.33 |
| 2010 | 626,444,358 | 14,433,665 | 612,010,693 | 2.30 |
| 2011 | 669,882,711 | 7,737,266 | 662,145,445 | 1.16 |
| 2012 | 601,849,846 | 15,275,772 | 586,574,074 | 2.54 |
| 2013 | 734,815,631 | 10,165,017 | 724,650,614 | 1.38 |
| 2014 | 800,653,862 | 8,141,807 | 792,512,055 | 1.02 |
| 2015 | 759,827,039 | 22,695,550 | 737,131,489 | 2.99 |
| 2016 | 1,061,161,682 | 18,388,894 | 1,042,772,788 | 1.73 |
| 2017 | 596,625,591 | 13,535,661 | 583,089,930 | 2.27 |
| 2018 | 809,737,525 | 23,568,973 | 786,168,552 | 2.91 |
| 2019 | 807,023,638 | 26,784,212 | 780,239,426 | 3.32 |
| 2020 | 844,704,939 | 15,399,858 | 829,305,081 | 1.82 |
| 2021 | 941,960,578 | 18,075,270 | 923,885,308 | 1.92 |
| 2022 | 1,034,036,087 | 21,645,338 | 1,012,390,749 | 2.09 |
| 2023 | 853,753,333 | 24,008,794 | 829,744,539 | 2.81 |

**Table S4: Funding Summary by Institute aggregated from 2008-2023**

| **Institute** | **CIHR Total Operational Funding Envelope**  **(CAD)** | **Total Operational Pain Funding (CAD)** | **Total Funding for All Other research Areas (CAD)** | **Percentage of Pain Funding** |
| --- | --- | --- | --- | --- |
| Aging | 642,994,216 | 4,340,521 | 638,653,695 | 0.68 |
| Cancer Research | 1,482,279,414 | 10,468,753 | 1,471,810,661 | 0.71 |
| Circulatory and Respiratory Health | 1,274,154,797 | 3,884,508 | 1,270,270,289 | 0.30 |
| Gender and Health | 210,332,937 | 11,326,508 | 199,006,429 | 5.39 |
| Genetics | 1,040,910,437 | 2,419,506 | 1,038,490,931 | 0.23 |
| Health Services and Policy Research | 941,513,499 | 26,072,404 | 915,441,095 | 2.77 |
| Human Development, Child and Youth Health | 831,760,511 | 22,611,476 | 809,149,035 | 2.72 |
| Indigenous Peoples' Health | 365,312,606 | 1,939,164 | 363,373,442 | 0.53 |
| Infection and Immunity | 1,522,555,531 | 3,162,725 | 1,519,392,806 | 0.21 |
| Musculoskeletal Health and Arthritis | 567,137,897 | 64,637,588 | 502,500,309 | 11.40 |
| Neurosciences, Mental Health and Addiction | 1,756,865,750 | 98,118,070 | 1,658,747,680 | 5.58 |
| Nutrition, Metabolism, and Diabetes | 760,793,406 | 3,912,649 | 756,880,757 | 0.51 |
| Population and Public Health | 602,549,342 | 3,258,166 | 599,291,176 | 0.54 |
| Unable to Allocate | 105,752,893 | 0 | 105,752,893 | 0.00 |
